# The Case of Miss Buchanan

**Published:** 1888-01-07

**Authors:** 


### THE CASE OF MISS BUCHANAN.

WE insert the following inquiries at the request of the mother, who has certainly no fair ground of complaint against us, for the reasons stated in paragraph 1. They are—

Is it usual, when writing to the Chairman of the Committee of Visitors of the County Asylums, and to the Lunacy Commissioners and Home Secretary, to expect a reply to questions asked in courteous communications? Is it usual to refuse a personal attendance by parents at a committee meeting in a county asylum?

1. The statement which we published about this case was taken from the last report of the Commissioners in Lunacy to the Lord Chancellor. At the time of publishing the extract we gave our authority for it, and do not think we are in any sense called upon to withdraw it, more especially as it was the patient's mother who first wrote to us in the matter, and but for her letters the extract would never have appeared. Should the Commissioners in Lunacy at any time modify their views of the case, or should a court of justice alter the aspect of those published proceedings, we shall, of course, be pleased to correct any impression which the appearance of the extract in our columns may have caused.

2. (*a*) The Committee of Visitors can make any rules for the government of their asylum not inconsistent with existing Acts of Parliament, and they are nowhere required to see friends of patients personally or answer inquiries; but our experience would lead us to believe that the Visiting Magistrates of our county asylums are extremely courteous to all, and never refuse interviews or answers without good reason. (*b*) We believe the Commissioners in Lunacy are both prompt and courteous in their answers. (*c*) We have not had much experience with the Home Secretary's office, but we think that, if somewhat slow in their replies, they also are invariably courteous.

We have already devoted too much space to this subject, and must be excused for declining to insert any more letters upon it.

